# The Population Explosion—A Personal Opinion

**Published:** 1971-07

**Authors:** J. C. Briggs

**Affiliations:** Consultant Pathologist, Frenchay Hospital, Bristol


### The Population Explosion
### A Personal Opinion

**J. C. Briggs, M.R.C.Path.**

**Consultant Pathologist, Frenchay Hospital, Bristol.**

We, in and around Bristol, live in a pleasant part of a pleasant land. In a very few years this could be changed with the "natural" increase of the city and it's suburbs. The population of the Bristol Clinical Area in 1951 was 699,525; in 1968 it was 803,824. Excluding any possible development of Severnside the projected estimates for 1981 and 2001 are 898,000 ± 11,000 and 1,041,000 ± 36,000 (1).

Many people in this and other countries are desperately concerned about the present population explosion. Some countries are doubling their population in twenty five years or so. Brazil is a case in point, a country which most of us would consider to contain a large percentage of people who live below the poverty line. These people fight to improve their lot, but, because of the population increase, within the next twenty five years there will have to be a doubling of schools, hospitals, houses, policemen, imports, exports etc., just to keep them in their present state of misery. Effort on this scale is not technologically possible, let alone the effort required to raise the general standard.

Such figures tend to mean little or nothing. Their generality and the remoteness of the country prevents comprehension. A more understandable viewpoint is perhaps reached when one realises that the annual increase of world population in 1969 (71 million) exceed the combined total killed in both World Wars (1914-18, 10 million; 1939-45, 55 million) (2). The admitted number of deaths in the Pakistan cyclone disaster (200,000) was replaced in one day when looked at in a world wide context.

Even when expressed in such terms the problem seems remote. Is it? I think not. The figures for the clinical area show that we are also having our own population explosion. Along with this comes the inevitable concrete upsurge to house the increase and the inevitable destruction of our environment by this concrete and the waste products of human existence (already the water in this hospital at times contains fourteen times or more than the W.H.O. recommended level of phenols (3)). To what end? As far as I can see, merely to have more people to generate more people. The concept horrifies me and I wonder into what sort of world I have brought my two children.

What can be done? Confronted by such thoughts most of my acquaintances have nothing to offer. Surely, as one of the most educated branches of the community, we must have some thoughts? As doctors we are in a unique position to take positive action—people are our concern.

Here are some suggested lines of action. They are not all my idea by any means:—

1) Read about population problems. Get to know them and go and bore your friends with them (Prince Phillip's suggestion). Those of you who know Councillors can plea for more family planning help from the Corporation. Make a noise. Write to your M.P.

2) Talk to your patients and offer contraceptive help. Point out that more than two children per family increases the population.

3) Persuade chemists to display contraceptive goods.

4) Refer patients for vasectomy on the N.H.S. (To my mind a country which offers a free abortion service but virtually no free contraception has its priorities wrong. Vasectomy for medical reasons can be performed on the N.H.S.).

5) If you are a surgeon—perform one or two vasectomies per list.

6) Have a more liberal view about abortion. Help to procure them.

As you will have gathered I feel strongly about this problem. Some people would regard me as a fanatic and an underminer of our basic morals. My outlook is summed up by John Stuart Mill in some very unfanatical words (4).

"There is room in the world, no doubt, and even in old countries, for a great increase of population, supposing the arts of life to go on improving, and capital to increase. But even if innocuous, I confess I see little reason for desiring it. The density of population necessary to enable mankind to obtain, in the greatest degrees, all the advantages both of co-operation and of social intercourse, has, in all the more populous countries, been attained. A population may be too crowded, though all be amply supplied with food and raiment. It is not good for a man to be kept perforce at all times in the presence of his species. A world from which solitude is extirpated is a very poor ideal. Solitude, in the sense of being often alone, is essential to any depth of meditation or of character; and solitude in the presence of natural beauty and grandeur, is the cradle of thoughts and aspirations which are not only good for the individual, but which society could ill do without. Nor is there much satisfaction in contemplating the world with nothing left to the spontaneous activity of nature; with every rood of land brought into cultivation, which is capable of growing food for human beings; every flowery waste or natural pasture ploughed up, all quadrupeds or birds which are not domesticated for man's use exterminated as his rivals for food, every hedgerow or superfluous tree rooted out, and scarcely a place left where a wild shrub or flower could grow without being eradicated as a weed in the name of improved agriculture. If the earth must lose that great portion of it's pleasantness which it owes to things that the unlimited increase of capital and population would extirpate from it, for the mere purpose of enabling it to support a larger, but not a better or happier population, I sincerely hope, for the sake of posterity, that they will be content to be stationary, long before necessity compels them to do it."

When Mill wrote that, the population of Britain was 20 millions and there were no motor cars.

**References**

(1) Population projections for the Bristol Clinical Area for the years 1981 and 2001 S.W.R.H.B., 4th March, 1969.

(2) Lancet. September 12th, 1970.

(3) Dr. G. R. Philpot. Personal communication.

(4) Quoted in "Why Britain needs a population policy". The Conservation Society, 1969.

